# Apparent Interfacial Tension Effects in Protein Stabilized Emulsions Prepared with Microstructured Systems

**DOI:** 10.3390/membranes7020019

**Published:** 2017-03-25

**Authors:** Carme Güell, Montserrat Ferrando, Alexandre Trentin, Karin Schroën

**Affiliations:** 1 Departament d’Enginyeria Química, Universitat Rovira i Virgili, Avda. Països Catalans 26, Tarragona 43007, Spain; montse.ferrando@urv.cat (M.F.); atrentin@gmail.com (A.T.); 2 Food Process Engineering Group, Wageningen University, Bornse Weilanden 9, 6708 WG Wageningen, The Netherlands; karin.schroen@wur.nl

**Keywords:** emulsion, premix membrane emulsification, microfluidic devices, proteins, interfacial tension

## Abstract

Proteins are mostly used to stabilize food emulsions; however, production of protein containing emulsions is notoriously difficult to capture in scaling relations due to the complex behavior of proteins in interfaces, in combination with the dynamic nature of the emulsification process. Here, we investigate premix membrane emulsification and use the Ohnesorge number to derive a scaling relation for emulsions prepared with whey protein, bovine serum albumin (BSA), and a standard emulsifier Tween 20, at various concentrations (0.1%, 0.5%, 1.25% and 2%). In the Ohnesorge number, viscous, inertia, and interfacial tension forces are captured, and most of the parameters can be measured with great accuracy, with the exception of the interfacial tension. We used microfluidic Y-junctions to estimate the apparent interfacial tension at throughputs comparable to those in premix emulsification, and found a unifying relation. We next used this relation to plot the Ohnesorge number versus P-ratio defined as the applied pressure over the Laplace pressure of the premix droplet. The measured values all showed a decreasing Ohnesorge number at increasing P-ratio; the differences between regular surfactants and proteins being systematic. The surfactants were more efficient in droplet size reduction, and it is expected that the differences were caused by the complex behavior of proteins in the interface (visco-elastic film formation). The differences between BSA and whey protein were relatively small, and their behavior coincided with that of low Tween concentration (0.1%), which deviated from the behavior at higher concentrations.

## 1. Introduction

Many food emulsions are prepared by standard homogenization techniques such as high-pressure homogenizers, colloid mills, or rotor stator systems, and the stabilizers of choice are proteins. Microstructured systems (membranes, microsieves or microfluidic devices) used for the production of emulsions are claimed to have lower energy requirements and higher control over the droplet size distribution than conventional equipment [1,2,3,4], and are therefore interesting alternatives for more traditional techniques that are much higher in energy demand.

Membranes can be used in two operation modes: direct membrane emulsification (cross-flow membrane emulsification) and premix membrane emulsification. In direct membrane emulsification, the to-be-dispersed phase flows through the membrane and the cross-flowing continuous phase shears of the droplets [1,5,6,7]. In premix membrane emulsification, a coarse emulsion is pressed through the membrane (sometimes several times) therewith applying shear to the droplets, and achieving a reduction of the droplet size [3].

In their review, Nazir et al. [8] state that premix membrane emulsification is better suited to produce relatively monodisperse emulsions at higher dispersed phase fractions than direct membrane emulsification because monodispersity of the latter emulsion is negatively influenced by the repeated cross-flow required for higher concentrations. Although the size of the droplets produced with premix emulsification is not as monodisperse as for direct cross-flow emulsification of dilute emulsions, for concentrated emulsions the monodispersity is very acceptable, which may make the technique interesting for industrial application. For an updated review on the applications of premix membrane emulsification to produce single and double emulsions the reader is referred to a recent paper from Güell et al. [9], and a review by Vladisavljevic and co-workers that comprises all emulsification techniques based on microstructures [3].

Microfluidic devices of different geometries have been used to investigate droplet formation under shear-based conditions, and various scaling relations have been derived [10,11,12,13,14,15]. In general, droplet formation depends on various parameters that have been reviewed for shear-base emulsification in microfluidic devices, but not for pre-mix emulsification. Based on what is reported for shear based systems it is expected that surface properties, transmembrane pressure, dispersed phase fraction, and continuous phase viscosity play important roles in premix emulsification, and besides that the number of cycles is expected to affect the droplet size as reported for emulsification with metal sieves [16].

Regarding droplet break-up mechanisms Nazir et al. [8] state that even though it is assumed that shear forces are responsible for droplet break-up, it is not clear how these forces operate during premix emulsification. In another source, in which microfluidic devices are used to visualize droplet break-up during premix emulsification, it is shown that various droplet formation mechanisms may be operating simultaneously [17]:

1. Localized shear forces: break-up due to the shear forces exerted on a droplet coming close to a branching channel, or due to divergent flow in both legs of a branching.
2. Interfacial tension effects: break-up due to deformation inside the channel because of the channel geometry.
3. Steric hindrance between the droplets: droplets accumulating before the membrane can influence each other and induce break-up.

From the above, it is clear that it will not be straight forward to capture all effects in one dimensionless number; here we consider three, Capillary, Weber and Ohnesorge number [18], and we compare their predictive power.

The Capillary number (*Ca*) is the ratio between the viscous force exerted by the continuous phase on a droplet and the interfacial tension force keeping the droplet connected (Equation (1)).

*Ca* = (*η_c_ v_c_*)/*γ* (1)

where *η_c_* is the viscosity of the continuous phase, *v*_c_ is the velocity of the continuous phase and *γ* is the interfacial tension between the dispersed phase and the continuous phase.

The Weber number (*We*, Equation (2)) is the ratio of the inertia forces and surface tension forces and is used to predict the disruption of an interface under strong inertial forces.

*We* = (*ρ_c_ v*_c_^2^*d*_32_)*/γ* (2)

where *ρ_c_* is the density of the continuous phase, *d*_32_ is the Sauter diameter of the droplets.

The Ohnesorge number (*Oh*, Equation (3)) relates the viscous, inertia, and surface tension forces and can be used to compare the relative importance of those forces during emulsification processes.

(3) $$Oh=\frac{\sqrt{We}}{Re}$$

where *Re* is the Reynolds number.

In all three dimensionless numbers, the interfacial tension is an important parameter that is difficult to assess at time scales as they occur during emulsification processes. Its value is influenced by the emulsifier used, mass transfer to the interface [19], and binding to the interface, the latter being very important for the formation of visco-elastic (protein) films [20,21]. The use of emulsifiers has two major effects: (1) lower the interfacial tension between the dispersed and the continuous phase therewith facilitating droplet break-up and (2) provide stability to the formed droplets against coalescence.

During droplet formation, the interfacial tension will at least temporarily deviate from its value at adsorption equilibrium [19,22,23,24], and may be even different at various locations on the interface (e.g., tip streaming) [25]. Steegmans and co-workers [26] have developed a methodology that to the best of our knowledge is the only technology available to investigate interfaces at the expansion rates applied during premix emulsification. Later, the method was further established by Muijlwijk, showing that it can be used for various emulsifiers [19,22]. The method allows estimation of the apparent interfacial tension based on a relationship between the capillary number and the surface area of the formed droplets in a microfluidic Y-junction.

For simple surfactants this method was successfully used, here we use it for the first time to assess proteins. The emulsifying properties of proteins are mainly related to the rate of adsorption at the oil/water (O/W) interface, the amount of adsorbed protein, the extent of reduction in interfacial tension, and the ability to form a cohesive and continuous film. The current paper will not address any changes in interfacial behavior due to e.g., oxidation [21], strong shearing stress [27], or random [28], or specific interaction with other components (layer by layer) [29]. It is important to mention that we expect that direct and premix membrane emulsification (ME) induce less of these side-effects [4], which may even be beneficial for emulsion stability [30].

The aim of this work is to study the production of O/W emulsions using proteins as emulsifiers. We chose two microstructured systems: (i) a premix membrane emulsification module for emulsion preparation [31] and (ii) a microfluidic Y-junction [26] for evaluation of the apparent interfacial tension in protein stabilized emulsions, using Tween 20 as a reference. The surface area of the droplets formed during emulsification in Y-junctions in combination with the known velocity and viscosity of the continuous phase was used to estimate apparent interfacial tension values. The apparent interfacial tension results are used as input for a dimensionless plot for premix membrane emulsification based on the Ohnesorge number. To be complete, droplet formation times in both techniques are much faster than in spontaneous emulsification techniques and scaling relations derived in that field cannot be used [32].

## 2. Materials and Methods

The premix emulsification and Y-junction experiments were carried out in two locations (Tarragona, Spain and Wageningen, The Netherlands, respectively) and therefore the used chemicals are specific for a certain experiment as indicated in the respective sections; we apologize for the seemingly repetitive description.

### 2.1. Premix Membrane Emulsification

#### 2.1.1. Materials

O/W emulsions were prepared using hexadecane, >99% (H-6703—From Sigma-Aldrich, Madrid, Spain) 10% *v*/*v*, as the dispersed phase. MilliQ water was used as continuous phase, containing 0.1%, 0.5%, 1.25% and 2% (*w*/*w*) Tween 20 (polyoxyethylene sorbitan monolaurate, from Sigma-Aldrich, Spain), 0.25%, 0.5% and 1% (*w*/*w*) BSA (albumin bovine/fraction V, from Acros Organics, Barcelona, Spain) or 0.25%, 0.5% and 1% (*w*/*w*) whey protein (BiPro, lot n° SE034-7440-b, from Davisco Foods, La Sueur, MN, USA). A phosphate buffer solution was used to adjust the pH to 7 before dissolving the proteins. No pH-adjustments made for emulsions prepared with Tween 20.

#### 2.1.2. Membranes and Membrane Characterization

Polymeric membrane discs of 25 mm diameter made of nitrocellulose mixed ester (MCE) (0.8 μm pore size, 150 μm thickness, and porosity 80%, Sterlitech Corporation ref. MCEB0847100SG, Kent, WA, USA) were used. The membrane diameter was approximately 21 mm, giving an effective filtration area of 3.46 × 10^−4^ m^2^.

#### 2.1.3. Experimental Set-Up and Procedure

O/W emulsions were prepared in two steps; first a coarse emulsion was prepared in a rotor-stator system (Ultra-Turrax^®^, model T18, IKA, Staufen, Germany) at 15,500 rpm for 2 min; typical droplet size was 12 μm for the Tween 20 emulsions, and 28 μm for the protein emulsions. This premix emulsion was then loaded into the reservoir and forced once through the membrane with nitrogen (at different transmembrane pressures ranging from 300 to 900 kPa) (step 2). The resulting fine emulsion was collected in a shake flask on a balance (A&D Fx-3000i, Brooklyn, NY, USA) connected to a computer. Mass was recorded every second, which was used to calculate the flux. Each experiment was carried out in triplicate; a new membrane was used for each repetition.

The typical water flux of the membrane is 33 kg/(m^2^·s) at 70 kPa. The flow rates during membrane emulsification were relatively low but stable and varied depending on the applied pressure and the type and concentration of emulsifier. Typical flux values for 2% Tween 20 were 10 to 48 kg/(m^2^·s) at 300 and 700 kPa, respectively, while for 0.25% and 0.5% BSA and whey protein the values were about 30 to 45 kg/(m^2^·s) at 600 and 900 kPa, respectively, and about 33 kg/(m^2^·s) at 900 kPa for 1% BSA.

#### 2.1.4. Emulsion Characterization

The mean droplet sizes (*d*_32_) of premix and final emulsions were measured with a Malvern Mastersizer 2000E (Worcestershire, UK) after emulsification was complete.

### 2.2. Microfluidic Y-Junctions

#### 2.2.1. Materials

Milli-Q water was used as the continuous phase. Anhydrous *n*-hexadecane with a purity of >99% (No. 296317, Sigma-Aldrich, Steinheim, Germany) was used as the dispersed phase. Tween 20 (polyoxyethylene sorbitan monolaurate, from Sigma-Aldrich, Spain), BSA (05473, fraction V, from Sigma Aldrich) or whey protein (BiPro, lot n° SE034-7440-b, from Davisco Foods) were used as emulsifiers and dissolved in MilliQ water. The concentrations of the proteins coincide with the ones used for premix ME (BSA and WP 0.25%, 0.5% and 1%), while for Tween 20 only a 2% solution was employed. A phosphate buffer solution was used to adjust the pH of all the solutions prepared with BSA and whey protein to 7. No pH adjustments were made for Tween 20 solutions.

#### 2.2.2. Experimental Setup

**Figure 1:**
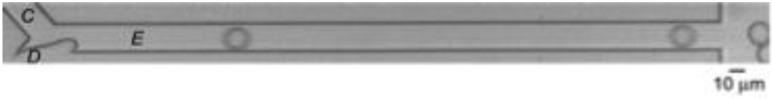
Image of the Y-junction microfluidic device showing the continuous phase channel (**C**), the dispersed phase channel (**D**) and the emulsion channel (**E**) (Figure reproduced by permission of John Wiley and Sons [license number 2711390361304]).

Microfluidic Y-Junction Devices: Borosilicate glass microfluidic devices with Y-junctions were produced by Micronit Microfluidics BV (Enschede, The Netherlands). The microfluidic device consists of a lower plate in which the Y channels are (chemically) etched and annealed to a top plate with inlets. The microchannels and collecting area have a uniform depth of 5 μm, which implies that the channels are flat (much wider than deeper). The angle between the channels for the continuous and dispersed phases was 97°, and the distance between the Y-junction and the collecting area was 0.46 mm (Figure 1). Detailed information on the operation of the Y-junction microfluidic devices can be found in the work of Steegmans [26].

Droplet-Formation Experiments: The Y-junction device was operated in the appropriate holder (No. 4515, Micronit Microfluidics BV, Enschede, The Netherlands). The continuous and dispersed phases entered the device via two separate fused silica capillaries of approximately 13 cm length with an inner diameter of 150 μm (Polymicro Technologies, Phoenix, AZ, USA). Each fused silica capillary was connected to a 2.5 mL stainless steel 1/16” Swagelock syringe. Swagelock connector (Alltech, Breda, The Netherlands), 9 cm PEEK tubing with an inner diameter of 1.6 mm (Alltech, Breda, The Netherlands), and a PEEK union assemblage with a capillary sleeve (Upchurch Scientific, Oak Harbor, WA, USA). The flow rate of the continuous and dispersed phases was controlled with syringe pumps (PHD4400 (continuous phase) and PHD22/2000 (dispersed phase), Harvard Apparatus, Holliston, MA, USA). Droplet formation was recorded using a high-speed camera (MotionPro HS-4, Redlake, Tallahassee, FL, USA) connected to an inverted transmitted light microscope (Axiovert 200, Carl Zeiss, Sliedrecht, The Netherlands). The formation of at least 25 subsequent droplets was recorded using frame rates between 500 and 10,000 s^−1^. Gain and exposure time were chosen such that the image quality was optimal.

#### 2.2.3. Analysis

Droplet Size: Droplet size was determined using Image-Pro Plus 4.5.0.29 (Media Cybernetics, Inc., Rockville, MD, USA). The diameter of each droplet was determined as the average taken from three subsequent frames from the exiting channel (round droplets). Ten subsequent droplets were measured; the droplet area used for further analysis was the average of these 10, which is an appropriate method to assess uniform droplets in our lab.

**Figure 2:**
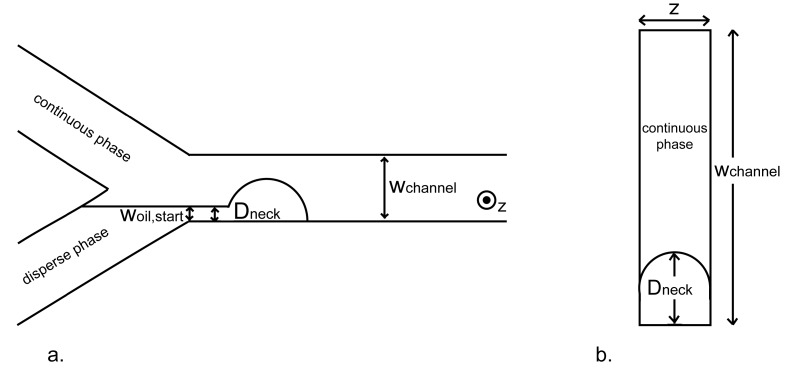
(**a**) Outline of the quantities defined in the second-to-last frame before droplet detachment and (**b**) cross-section of the downstream channel with the oil neck. In both images, the dimensions and the angle are not to scale. Reprinted with permissions from [26]. Steegmans, M.L.J.; Warmerdam, A.; Schroën, K.G.P.H.; Boom, R.M. Dynamic interfacial tension measurements with microfluidic Y-junctions. *Langmuir* **2009**, *25*, 9751–9758. Copyright 2009 American Chemical Society.

Droplet-Formation Quantities: Two characteristic quantities were defined in the second-to-last frame before droplet detachment: width of the dispersed phase at the corner of the Y junction (*w*_oil,start_) and width of the thinnest point of the hexadecane filament keeping the droplet attached to the hexadecane bulk (neck) (*D*_neck_) (Figure 2a). The quantities were manually determined with Image-Pro Plus 4.5.0.29. For each quantity, the average of 10 droplets was taken.

Flow Rates at the Y-Junction: The actual flow rates at the Y junction were found to differ from the flow rates set on the pump, probably because of the large pressure drop in the microfluidic device [26]. The continuous phase flow rate ϕ_c_ at the Y junction was determined from the velocity of the droplets in the downstream channel, assuming the droplet velocity to be equal to the velocity of the continuous phase. Using Image-Pro Plus 4.5.0.29, the length between the downstream corners of the Y junction and the left side of the droplet was manually determined in the third frame after detachment and in the frame after which the droplet moved outside the image. Subsequently, the velocity followed from the difference in length divided by the accompanying time between the two frames.

## 3. Results and Discussion

### 3.1. Droplet Size Reduction after Premix Membrane Emulsification

**Figure 3:**
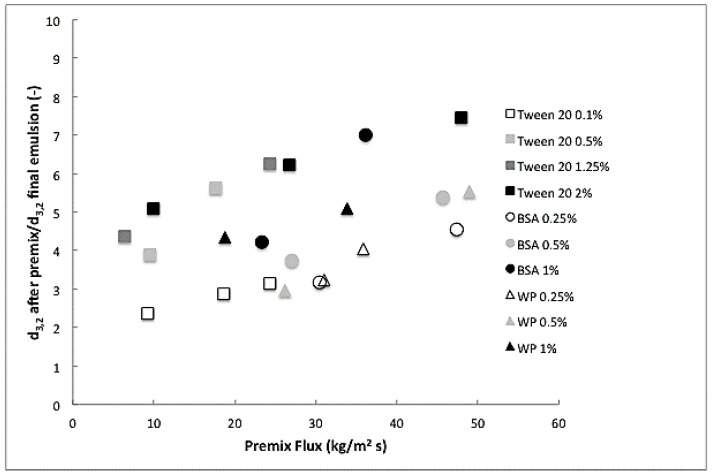
Droplet size reduction (diameter droplets after premix/diameter droplets final emulsion) as function of the premix flow rate.

Whey protein (WP), BSA, and Tween 20 at different concentrations were employed to obtain 10% (*w*/*w*) hexadecane/water emulsions. A coarse emulsion was produced using rotor stator mixing and then this coarse emulsion was passed through a membrane. Since the starting emulsions did not have the same size (measured values are given in Supplementary Materials, Table S1), we here compare the results based on the reduction in size that was achieved (*D*_after premix_/*D*_final_), as illustrated in Figure 3 for different premix fluxes.

The general trend, regardless of the emulsifier used, is that increasing the premix flow increases the reduction in droplet size, i.e., leads to smaller droplets that are smallest for the highest concentrations used. The smallest droplet found for Tween 20 is about two times the mean pore size of the membrane (0.8 μm), which is considered the limit for premix emulsification. There is a notable difference between proteins and Tween 20; clearly the surfactant is more efficient in size reduction than the proteins. The highest Tween 20 concentrations are all above the critical micelle concentration, and do not show a difference in size reduction. When taking the equilibrium interfacial tension as an argument (Table 1), as often done in literature, this is expected. On the other hand, 1% BSA was also very effective in reducing the droplet size, and that was not expected based on the static interfacial tension, which is quite high. From this, we conclude that dynamic interfacial tension effects need to be taken into account.

To investigate this further, we used the microfluidic Y junctions to obtain estimates for the apparent interfacial tension. In the final section, these values are implemented in the dimensionless numbers presented earlier to obtain scaling relations for pre-mix membrane emulsification.

### 3.2. Apparent Interfacial Tension Measurement

**Figure 4:**
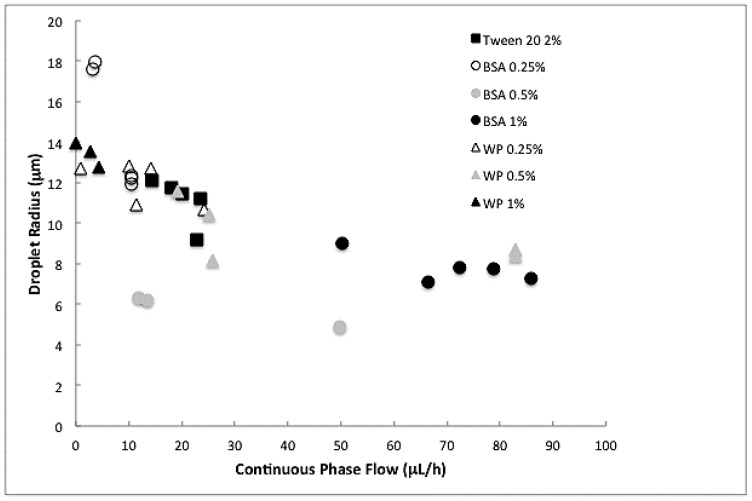
Radius of hexadecane droplets versus continuous phase flow in Y-junctions using Tween 20, BSA, or whey protein at different concentrations.

Before going into detail, we first show the general picture obtained from the Y-junctions. Figure 4 shows the droplet radius of hexadecane droplets generated in the microfluidic Y-junction as a function of the continuous phase flow for the various emulsifier concentrations used. Regardless of the emulsifier, an increase in the continuous phase flow (increase in shear force) caused a decrease in droplet size, which is in line with [22].

Muijlwijk and co-workers [22] found a unifying model in the dripping and transition regimes that can be used to predict the apparent interfacial tension for a range of surfactants [19], but that did not hold in the squeezing regime. It is important to note that the Y-junctions are difficult to control when using proteins compared to standard emulsifiers. In some cases, droplets larger than the channel were formed (squeezing mechanism) and these data points needed to be discarded from our analysis.

**Figure 5:**
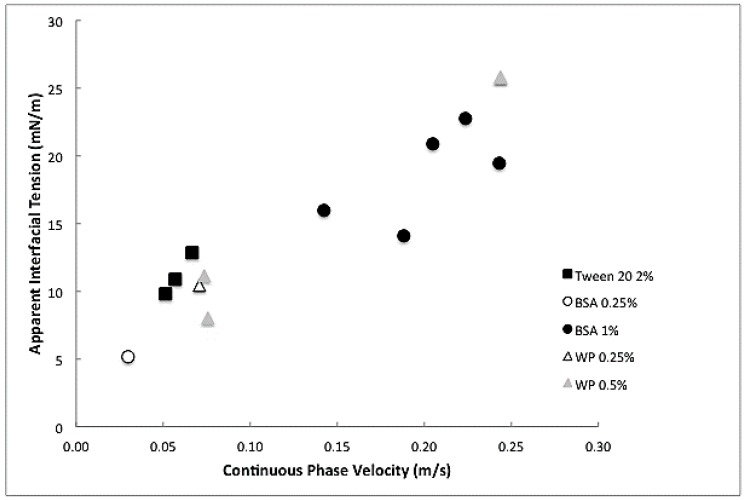
Apparent interfacial tension versus the continuous phase flow rate for hexadecane/water droplets using Tween 20, BSA, or whey protein at different concentrations.

For those data points measured in the dripping and transition regime, the interfacial tension played a role on droplet formation in Y-junctions that can accurately be described with a Capillary number [22,26], unlike premix emulsification, as will be discussed later. The droplet radius obtained from the recorded movies was used in a correlation developed by Steegmans [26], from which the apparent interfacial tension can be derived using Equation (1), since the viscosity and the velocity of the continuous phase are both known (Figure 5).

The apparent interfacial tension was lower at low continuous phase velocity and increases at higher velocity. This was as expected, since at higher continuous flow rates the interface expands faster. Surprisingly, the bulk surfactant concentration did not influence the actual apparent interfacial tension that much, which could be indicative of interfaces close to saturation, and the protein and Tween data seemed to practically coincide (Figure 5), as will be discussed later. Most importantly, a fairly simple relation is now available to link the throughput (the flux measured during emulsification) with the apparent interfacial tension, which is very convenient when establishing windows of operation for emulsification processes (as will be done in the next section).

The apparent interfacial tension was a balance between the expansion rate and the amount of surfactant that adheres to the surface during the expansion time. Surfactant adhesion consisted partly of diffusion/transport and partly of binding to the surface, both with their characteristic times. Although the flow in the microchannels seemed laminar based on the overall Reynolds number, locally large velocity differences might occur when the filament that keeps the droplet connected brakes. In the work of Muijlwijk, it was demonstrated that mass transfer is dominated by convective effects in the microchannels [22], leading to a fast reduction in interfacial tension.

Even more surprisingly, the values found for the simple surfactant Tween 20, seemed to be higher than for protein at the same continuous phase flow velocity, while the equilibrium interfacial tensions measured in a volume droplet tensiometer was much lower for Tween 20 than for any of the proteins. This is only possible if the protein has different properties in a shear situation compared to a diffusion situation as used in the droplet volume tensiometer (fully diffusion based, and equilibrium conditions). The unexpected low values for proteins could indicate that they underwent conformational changes that affected their surface properties. Bos and van Vliet [20] have pointed out that once proteins are absorbed, their conformation may change considerably at hydrophobic surfaces as present in O/W emulsions, and the conformational changes will depend on the nature of the protein. So-called flexible proteins (e.g., casein) are more easily deformed than so-called hard proteins, like BSA or whey proteins as employed in this study. In so called Lissajous plots, the dynamic shear thinning interfacial behavior of whey protein has been demonstrated, and this may also have contributed to the observed behavior [21,34].

In summary, structural changes in the protein possibly leading to visco-elastic films are expected to have caused the relatively low apparent interfacial tensions, which may even be reflected in the image parameters *w*_oil,start_ (width of the dispersed phase at the corner of the Y junction) and *D*_neck_ (filament keeping the droplet attached to the hexadecane bulk). In the Supplementary Materials Table S2, the values of these two parameters for each emulsifier and velocity of the continuous phase are shown. We can observe that *D*_neck_ and *w*_oil,start_ are the same when the droplets were produced using Tween 20 as was found for other standard emulsifiers. However, for the proteins this is not always the case and this could indicate a local difference in interfacial tension possibly caused by the visco-elastic films formed by the proteins.

### 3.3. Dimensionless Numbers

When using either static or apparent interfacial tension values, we found that neither the Capillary number nor the Weber number could describe/summarize our results appropriately. The mechanisms behind premix membrane emulsification are more complex as expected, and therefore we used the Ohnesorge number in which various mechanisms are combined.

We used a comparative measure called P-ratio (applied pressure over the Laplace pressure of the feed droplet; i.e., the actual applied pressure over the minimum pressure needed to deform a droplet based on the static interfacial tension related to the concentration used; Table 1), and plotted this as function of the Ohnesorge number (Oh). To make the Ohnesorge number (Equation (3) suitable to describe premix ME we have modified it to incorporate the membrane thickness and the diameter of the pore as shown in Equation (4)

(4) $$Oh^{*}=\frac{\eta_{c}\sqrt{d_{32}}}{\sqrt{\rho_{c}\gamma d_{m}\lambda_{m}}}$$

where Oh* is the modified Ohnesorge number, *d_m_* is the pore size diameter of the membrane and *λ_m_* is the membrane thickness. For the density and viscosity, the values of the continuous phase were taken (water), which were good assumptions for 10% hexadecane/water emulsions. For the interfacial tension, the apparent interfacial tension values were calculated based on the throughput in the membranes when compared to those used in the Y-junctions. Basically, the range between 0 and 30 μL/h corresponds to 0 to 0.05 m/s.

**Figure 6:**
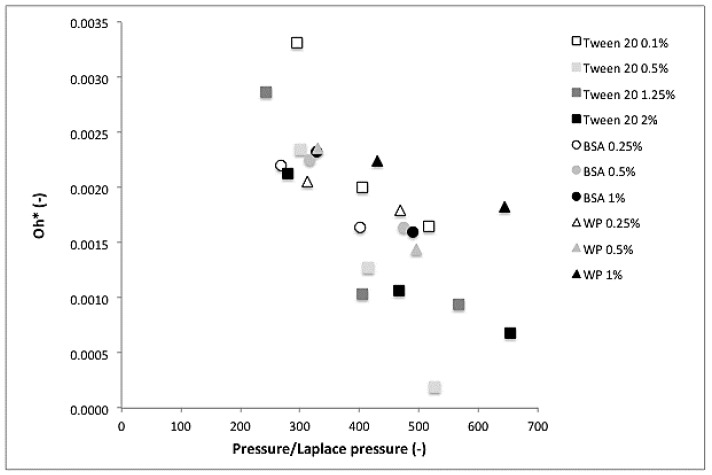
Relationship between the Oh* number and the pressure ratio for the premix membrane emulsification (ME) experiments with different concentrations of Tween 20, BSA and whey protein based on the apparent interfacial tension calculated from the Y-junction experiments.

In Figure 6, the calculated values of the Oh* number for the emulsions produced by premix ME are presented as a function of the P-ratio. In all cases, the Oh* number decreased with increasing applied pressure, which is indicative of greater size reduction at higher pressures. There is a clear difference between Tween 20 and proteins, with Tween 20 being the more efficient at size reduction at higher applied pressures. Only when present at low concentration (0.1%), the Oh* number was similar to that found for the proteins. This can indicate that surfactant transport toward the interface is rather slow, resulting in relatively high interfacial tensions, or that re-coalescence occurs, leading to increased droplet size. When comparing BSA and whey protein, the data points for whey protein seem to be slightly higher than their counterparts for BSA measured at the same concentration. This could indicate that using more pure proteins would be beneficial for emulsion preparation, but in all honesty, we find the difference too small to speculate any further.

The presented dimensionless plot covers essential process conditions for pre-mix membrane emulsification (pressure, viscosity, density, membrane thickness, and pore size) and includes the equilibrium and apparent interfacial tension. It links all these parameters for the first time, and is in our opinion a good indicator for the generated droplet size.

## 4. Conclusions

The results that were obtained for premix membrane emulsification can be summarized using a modified Ohnesorge number, which accounts for viscous, inertia, and interfacial forces, and a pressure ratio that relates the applied pressure and the Laplace pressure of the droplet. The results obtained for protein containing emulsions and Tween 20 showed the same trends when using the apparent interfacial tension as measured with microfluidic devices as input. The differences may be caused by visco-elastic film formation when using proteins as ingredients. The presented scaling plot will facilitate (protein-based) membrane emulsification process design.

## Figures and Tables

**Table 1:** Equilibrium oil/water interfacial tension measurements for the emulsifiers used, as obtained by the Drop Volume Method [33].

Oil Phase	Continuous Phase	Concentration (*w*/*w* % of Cont. Phase)	Interfacial Tension (mN/m)
Hexadecane	Tween 20	0.10	4.2
		0.50	3.7
		1.25	3.4
		2.00	3.1
	BSA	0.25	14.6
		0.50	13.6
		1.00	13.6
	Whey protein	0.25	12.9
		0.50	12.5
		1.00	10.3

## Supplementary Materials

Supplementary File 1
