# Supplementary material for: Apparent Interfacial Tension Effects in Protein Stabilized Emulsions Prepared with Microstructured Systems
Source: Membranes (Basel). 2017 Mar 25;7(2):19. doi: 10.3390/membranes7020019 (PMC5489853; doi:10.3390/membranes7020019)
Supplement: Supplementary File 1 [file membranes-07-00019-s001.pdf]

Supplementary Materials: Apparent Interfacial Tension Effects in Protein Stabilized Emulsions Prepared with Microstructured Systems

Carme Güell, Montserrat Ferrando, Alexandre Trentin and Karin Schroën

Table S1. Measured droplet diameters after premix and in the final emulsion obtained for Tween 20, BSA, and whey protein.

Emulsifier	Conc. (%)	Premix Flow (Kg/m² s)	Droplet Diameter D32 (µm)	
			D after Premix (µm)	D final Emulsion (µm)
Tween 20	0.1	9.16 ± 0.07	12.510 ± 0.789	5.308 ± 0.312
		18.56 ± 1.33		4.326 ± 0.258
		24.31 ± 0.23		3.987 ± 0.043
	0.5	9.47 ± 1.95	11.266 ± 0.122	2.895 ± 0.042
		17.59 ± 0.06		2.000 ± 0.037
		32.94 ± 0.42		0.665 ± 0.005
	1.25	6.44 ± 0.20	11.140 ± 0.445	2.5545 ± 0.029
		24.45 ± 4.68		1.779 ± 0.063
		28.19 ± 6.33		1.737 ± 0.011
	2	10.04 ± 1.33	11.591 ± 0.234	2.277 ± 0.079
		26.74 ± 0.28		1.857 ± 0.039
		47.98 ± 0.55		1.551 ± 0.011
BSA	0.25	30.49 ± 0.25	26.110 ± 0.728	8.192 ± 1.828
		47.38 ± 0.63		5.732 ± 0.238
	0.5	26.95 ± 5.52	28.771 ± 1.345	7.663 ± 0.257
		45.73 ± 14.62		5.328 ± 0.231
	1	23.35 ± 0.27	29.640 ± 0.692	7.001 ± 0.352
		36.11 ± 0.85		4.234 ± 0.224
WP	0.25	30.96 ± 0.05	26.952 ± 2.146	8.331 ± 3.886
		35.83 ± 0.33		6.696 ± 0.236
	0.5	26.17 ± 0.09	27.425 ± 0.896	9.230 ± 0.368
		49.04 ± 12.69		4.962 ± 0.790
	1	18.79 ± 1.27	29.426 ± 2.058	6.8032 ± 1.165
		33.91 ± 3.04		5.806 ± 0.071

Table S2. Values of D_{neck} and $w_{oil,start}$ measured during oil droplet formation in the Y-junctions with Tween 20, BSA or whey protein at different concentrations.

Continuous Phase	Cont. Phase Velocity (m/s)	D_{neck} (µm)	$w_{oil,start}$ (µm)
1% BSA	0.143	6.53	6.53
	0.188	4.73	4.73
	0.223	7.88	7.88
	0.243	8.17	8.17
	0.205	6.53	6.53
0.5% BSA	0.059	5.60	5.60
	0.052	4.90	4.90
	0.219	5.32	5.32

Table S2. *Cont.*

0.25% BSA	0.008	7.53	13.18
	0.009	5.65	13.18
	0.030	5.88	5.88
	0.030	8.17	8.17
	0.029	7.51	7.51
1% whey protein	0.007	8.12	N/A
	0.012	8.17	N/A
	0.021	4.08	8.49
0.5% whey protein	0.076	6.14	8.66
	0.244	8.35	8.35
	0.244	7.88	8.82
	0.074	5.95	9.61
	0.057	6.30	6.30
0.25% whey protein	0.033	6.30	6.30
	0.041	5.83	9.45
	0.003	6.30	7.56
	0.029	4.73	5.67
	0.071	8.66	6.12
2% Tween 20	0.040	6.53	6.53
	0.057	6.53	6.53
	0.065	8.18	8.18
	0.067	6.53	6.53
